# Tranexamic acid for treatment of women with post-partum haemorrhage in Nigeria and Pakistan: a cost-effectiveness analysis of data from the WOMAN trial

**DOI:** 10.1016/S2214-109X(17)30467-9

**Published:** 2018-01-19

**Authors:** Bernadette Li, Alec Miners, Haleema Shakur, Ian Roberts, WOMAN Trial Collaborators

**Affiliations:** a Department of Health Services Research and Policy, London School of Hygiene & Tropical Medicine, London, UK; b Clinical Trials Unit, London School of Hygiene & Tropical Medicine, London, UK

## Abstract

**Background:**

Sub-Saharan Africa and southern Asia account for almost 85% of global maternal deaths from post-partum haemorrhage. Early administration of tranexamic acid, within 3 h of giving birth, was shown to reduce the risk of death due to bleeding in women with post-partum haemorrhage in the World Maternal Antifibrinolytic (WOMAN) trial. We aimed to assess the cost-effectiveness of early administration of tranexamic acid for treatment of post-partum haemorrhage.

**Methods:**

For this economic evaluation we developed a decision model to assess the cost-effectiveness of the addition of tranexamic acid to usual care for treatment of women with post-partum haemorrhage in Nigeria and Pakistan. We used data from the WOMAN trial to inform model parameters, supplemented by estimates from the literature. We estimated costs (calculated in 2016 US$), life-years, and quality-adjusted life-years (QALYs) with and without tranexamic acid, calculated incremental cost-effectiveness ratios (ICERs), and compared these to threshold values in each country. Costs were assessed from the health-care provider perspective and discounted at 3% per year in the base case analysis. We did a series of one-way sensitivity analyses and probabilistic sensitivity analysis to assess the robustness of the results to parameter uncertainty.

**Findings:**

Early treatment of post-partum haemorrhage with tranexamic acid generated an average gain of 0·18 QALYs at an additional cost of $37·12 per patient in Nigeria and an average gain of 0·08 QALYs at an additional cost of $6·55 per patient in Pakistan. The base case ICER results were $208 per QALY in Nigeria and $83 per QALY in Pakistan. These ICERs were below the lower bound of the cost-effectiveness threshold range in both countries. The ICERs were most sensitive to uncertainty in parameter inputs for the relative risk of death due to bleeding with tranexamic acid, the discount rate, the cost of the drug, and the baseline probability of death due to bleeding.

**Interpretation:**

Early treatment of post-partum haemorrhage with tranexamic acid is highly cost-effective in Nigeria and Pakistan, and is likely to be cost-effective in countries in sub-Saharan Africa and southern Asia with a similar baseline risk of death due to bleeding.

**Funding:**

London School of Hygiene & Tropical Medicine, Pfizer, UK Department of Health, Wellcome Trust, and Bill & Melinda Gates Foundation.

## Introduction

Between 1990 and 2015, the global maternal mortality ratio declined from 385 deaths per 100 000 births to 216 deaths per 100 000 births but fell short of the 75% reduction called for in the Millennium Development Goals (MDG) framework.^1^ In 2015, the continued commitment to reducing maternal mortality was outlined in the Sustainable Development Goals (SDGs), which established a target of reducing the global maternal mortality ratio to less than 70 per 100 000 births by 2030.^2^ Post-partum haemorrhage, commonly defined as a blood loss of more than 500 mL within 24 h of giving birth, is a leading cause of maternal death, accounting for approximately 20% of maternal deaths globally.^3^ Interventions aimed at preventing or treating post-partum haemorrhage can have an important role in working towards the SDG target.

Tranexamic acid is a drug that reduces bleeding by inhibiting the breakdown of fibrin blood clots.^4^ The effect of tranexamic acid in reducing the risk of death from post-partum haemorrhage was shown in the World Maternal Antifibrinolytic (WOMAN) trial, a randomised, double-blind, placebo-controlled study that enrolled more than 20 000 women in 21 countries between March, 2010, and April, 2016.^5^ The burden of maternal deaths falls disproportionately on low-income and middle-income countries; sub-Saharan Africa and southern Asia account for almost 85% of global maternal deaths from post-partum haemorrhage.^3^ In resource-constrained settings, the decision to adopt a medicine for routine use in clinical practice should be informed not only by information about the clinical effectiveness of an intervention but also by information about its cost-effectiveness.^6^ We aimed to evaluate the cost-effectiveness of tranexamic acid for treatment of post-partum haemorrhage in Nigeria and Pakistan. Cost-effectiveness analyses at the country level are needed to reflect differences in both costs and the baseline risk of maternal death. We focus on Nigeria and Pakistan because they are among the countries with the highest number of maternal deaths annually^7^ and more than 50% of patients in the WOMAN trial were enrolled from these two countries.

**Research in context**

**Evidence before this study**

We searched the PubMed database to identify cost-effectiveness analyses of tranexamic acid published before June, 2017. Two searches were done, with the following search terms: cost effectiveness AND tranexamic acid (filter: humans); and cost effectiveness AND post-partum haemorrhage (filter: humans). Eligible studies had to include a comparative analysis of both costs and effectiveness and to report results in terms of an incremental cost-effectiveness ratio. We identified three studies that had evaluated the cost-effectiveness of tranexamic acid, two in bleeding trauma patients and one in patients who had elective surgery. We did not identify any previous studies that had assessed the cost-effectiveness of tranexamic acid for treatment of women with post-partum haemorrhage.

**Added value of this study**

The international, multicentre, randomised World Maternal Antifibrinolytic (WOMAN) trial, which enrolled over 20 000 patients between March, 2010, and April, 2016, showed that early administration of tranexamic acid reduces the risk of death due to bleeding in women with post-partum haemorrhage. This economic evaluation draws on data from the WOMAN trial to inform an assessment of the cost-effectiveness of tranexamic acid for treatment of women with post-partum haemorrhage in Nigeria and Pakistan, taking into account country-specific differences in both costs and baseline risk of mortality.

**Implications of all the available evidence**

Consistent with the results of previous cost-effectiveness analyses in other patient groups and settings, the findings presented in this study suggest that early administration of tranexamic acid in women with post-partum haemorrhage is cost-effective. This finding is important given the resource constraints faced by countries in sub-Saharan Africa and southern Asia, two regions with a disproportionate burden of global maternal deaths due to post-partum haemorrhage.

## Methods

### Model structure and comparators

**Figure 1:**
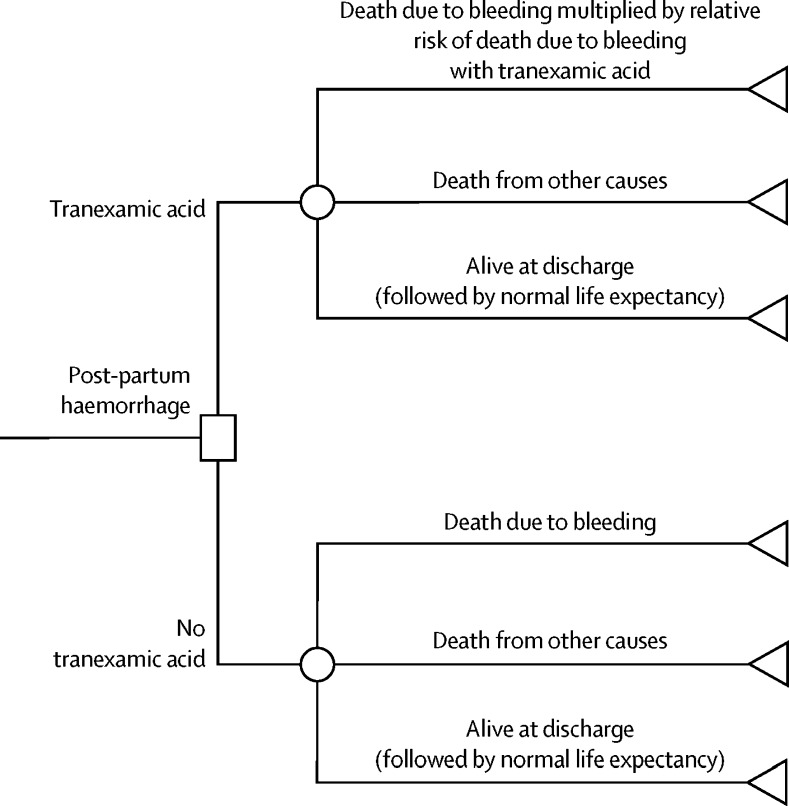
Decision tree Structure of the decision tree used to evaluate the cost-effectiveness of treating post-partum haemorrhage with and without tranexamic acid and showing where the effect of tranexamic acid in reducing the relative risk of death due to bleeding is applied in the model.

For this economic evaluation we developed a decision tree (figure 1) to evaluate the cost-effectiveness of treating post-partum haemorrhage with and without tranexamic acid using data from the WOMAN trial, supplemented by literature-based sources where necessary. In the model, following diagnosis of post-partum haemorrhage, there are three possible outcomes: death due to bleeding, death from other causes, or alive at discharge (or 42 days after randomisation if not yet discharged).^8^ Because of its mechanism of action, tranexamic acid is not expected to have any effect on deaths that are not related to bleeding^5^ and therefore these events are estimated separately in the model. For patients who were alive at discharge, the model assumes that their long-term survival is equivalent to the age-adjusted life expectancy of the general female population in each country.^9^, ^10^

In the WOMAN trial, tranexamic acid was administered by intravenous injection. Results of prespecified subgroup analyses showed that the effect of tranexamic acid in reducing the risk of death due to bleeding varied with time to initiation of treatment. Early treatment, defined as administration of tranexamic acid within 3 h of giving birth, substantially reduced the risk of death due to bleeding compared with placebo (risk ratio [RR] 0·69, 95% CI 0·52–0·91) whereas no reduction was observed when tranexamic acid was given after 3 h.^5^ This cost-effectiveness model therefore evaluated the effect of early treatment with tranexamic acid by focusing on the subgroup of patients who received treatment within 3 h of giving birth in the WOMAN trial.

The cost-effectiveness model was constructed with the software package TreeAge Pro 2017. Analyses of data from the WOMAN trial to inform the cost-effectiveness model were done in Stata, version 14.

### Study conduct

The WOMAN trial was done in accordance with good clinical practice guidelines of the International Conference on Harmonisation of Technical Requirements for Pharmaceuticals for Human Use (ICH).^11^ The consent procedures are described in detail in the protocol.^8^ The procedure at each site was approved by the relevant ethics committee and regulatory agencies. The trial is registered with the ISRCTN registry, number ISRCTN76912190 (Dec 8, 2008); ClinicalTrials.gov, number NCT00872469; and the Pan African Clinical Trials Registry, number PACTR201007000192283.

### Data inputs

The model reports outcomes in terms of both survival (in years) and quality-adjusted life-years (QALYs). Survival estimates were informed by data from the WOMAN trial. The baseline probability of death due to bleeding in each country was estimated from the placebo group (table 1), to which the relative risk of death for patients who received tranexamic acid was applied. To estimate the probability of death from other causes, we first used a χ^2^ test to assess the equality of proportions between the placebo and tranexamic acid groups and found no difference. Therefore, we calculated a single estimate of the probability of death from other causes that we applied to both groups in the cost-effectiveness model. We estimated time to death due to bleeding, time to death from other causes, and time to discharge by pooling data from both groups of the WOMAN trial after *t* tests confirmed that there were no differences between patients who received tranexamic acid and patients who received placebo.

**Table 1:** Data inputs to estimate survival in the cost-effectiveness model

		**Base case value**	Range (one-way sensitivity analysis)*	**Distribution (probabilistic sensitivity analysis)**
Baseline probability of death due to bleeding				
	Nigeria	2·79%	2·08–3·65	Beta: *r*=51; n=1831
	Pakistan	1·12%	0·72–1·66	Beta: *r*=24; n=2141
Relative risk (RR) of death due to bleeding with tranexamic acid given within 3 h		0·69	0·52–0·91	Lognormal: ln(RR) −0·370; SE[ln(RR)] 0·137
Baseline probability of death from other causes				
	Nigeria	0·76%	0·50–1·09	Beta: *r*=28; n=3694
	Pakistan	0·67%	0·45–0·97	Beta: *r*=29; n=4308
Time to death due to bleeding, years		0·0015	0·0007–0·0023	Gamma: mean 0·0015; SE 0·0004
Time to death due to other causes, years		0·0078	0·0051–0·0104	Gamma: mean 0·0078; SE 0·0013
Time to discharge, years		0·0093	0·0092–0·0095	Gamma: mean 0·0093; SE 0·0001
Post-discharge age-adjusted female life expectancy, years†				
	Nigeria	40·9	36·8–45·7	Normal: mean 40·9; SE 1·28
	Pakistan	49·2	45·9–52·2	Normal: mean 49·2; SE 1·02

All data are from the WOMAN trial,^5^ unless otherwise stated.

* For inputs derived from the WOMAN trial, the range reflects the 95% CI.

† Sources: WHO Global Health Observatory,^9^ Lopez et al (2000).^10^

A QALY is an outcome measure that combines mortality and morbidity into a single index, thereby recognising that health improvements can reflect gains in both quantity and quality of life.^12^ To estimate QALYs, the time spent in a given health state is multiplied by a quality-adjustment weight, also referred to as a health-state utility value. The conventional scale for health-state utility values ranges from 0 (death) to 1 (perfect or full health).^12^ In the WOMAN trial, health status was measured in patients who were alive at discharge (or 42 days after randomisation) by the doctor or midwife with the proxy version of the European Quality of Life 5 Dimensions-3 Level (EQ-5D-3L) questionnaire and converted to index scores via the value set for the UK (table 2). At present, no value sets are available for Nigeria and Pakistan. We observed no difference in index scores between patients who received tranexamic acid and patients who received placebo, and therefore the same value of 0·895 was applied to all patients for the period between randomisation and discharge.

**Table 2:** Data inputs for health-state utility values in the cost-effectiveness model

	**Base case value**	Range (one-way sensitivity analysis)*	**Distribution (probabilistic sensitivity analysis)**	**Source**
Utility value for patients alive at discharge	0·895	0·892–0·897	Beta: mean 0·895; SE 0·001	WOMAN trial^5^
Utility value for patients in hospital before death (any cause)	0·41	0·20–0·63	Beta: mean 0·41; SE 0·11	Alfirevic et al (2016)^14^
Utility value after discharge (general female population)	0·93	0·91–0·94	Beta: mean 0·93; SE 0·007	Kind et al (1999)^13^

* For inputs derived from the WOMAN trial, the range reflects the 95% CI.

For patients who were alive at discharge, we assumed a health-state utility value for the post-discharge period until death of 0·93, based on values for the UK general female population.^13^ For patients who died, a health-state utility value of 0·41 was obtained from a published systematic review of women undergoing emergency caesarean section requiring intensive care and was applied to the period between randomisation and death.^14^

We considered costs from the perspective of the health-care provider and included the cost of tranexamic acid plus the cost of administration,^15^, ^16^ and the cost of hospital stays (table 3).^17^ Tranexamic acid was administered as a 1 g dose by slow intravenous injection. Administration costs in the model took into account the cost of syringes and the cost of 10 min of nurse time derived from WHO-CHOICE region-specific personnel costs.^15^ As indicated in the protocol, if bleeding continued after 30 min or restarted within 24 h of the first dose, a second 1 g dose could be given.^8^ Approximately 30% of patients received a second dose of tranexamic acid. To calculate the cost of hospital stays, we multiplied the time between randomisation and discharge by country-specific estimates of cost per hospital bed-day based on WHO-CHOICE health service delivery costs, which estimate the hotel component of hospital costs, including personnel, capital, and food costs but excluding condition-specific treatments and diagnostic tests.^17^

**Table 3:** Data inputs to estimate costs in the cost-effectiveness model

		**Base case value**	Range (one-way sensitivity analysis)*	**Distribution (probabilistic sensitivity analysis)**	**Sources**
Mean number of doses of tranexamic acid administered		1·29	1·28–1·30	Gamma: mean 1·29; SE 0·005	WOMAN trial^5^
Cost per 1 g dose of tranexamic acid (US$)					
	Nigeria	29·84	4·30–34·00	NA	Hilton Pharma Ltd
	Pakistan	5·60	4·30–10·70	NA	Holy Family Hospital (Rawalpindi, Pakistan)
Cost of administration per dose of tranexamic acid: two syringes, 10 min nurse time (US$)					
	Nigeria	1·50	NA	NA	WHO-CHOICE,^15^ WHO^16^
	Pakistan	0·59	NA	NA	WHO-CHOICE,^15^ WHO^16^
Cost per hospital bed-day (US$)					
	Nigeria	24·77	23·74–32·03	NA	WHO-CHOICE^17^
	Pakistan	32·15	30·80–41·59	NA	WHO-CHOICE^17^
Proportion of patients requiring laparotomy (placebo)		1·08%	0·86–1·34	Beta: *r*=80; n=7408	WOMAN trial^5^
Proportion of patients requiring laparotomy (tranexamic acid)		0·55%	0·39–0·74	Beta: *r*=41; n=7475	WOMAN trial^5^
Cost of laparotomy (US$)					
	Nigeria	746	154–905	NA	University College Hospital (Ibadan, Nigeria)
	Pakistan	330	172–480	NA	Holy Family Hospital (Rawalpindi, Pakistan)

NA=not applicable.

* For inputs derived from the WOMAN trial, the range reflects the 95% CI.

In the model, we also considered the influence of clinical events that differed significantly between treatment groups and that were expected to incur additional costs for the health-care provider. For patients in the WOMAN trial who received treatment within 3 h of giving birth, no significant differences were observed between groups in the rates of brace suture, mechanical ventilation, transfusion, hysterectomy, administration of uterotonic drugs, or number of days spent in the intensive care unit (appendix). However, the rate of laparotomy was higher in the placebo group than in the tranexamic acid group; therefore, we took the cost of laparotomy into account in our analysis.

The model did not attempt to account for future health-care costs beyond the time horizon of the trial.^18^ Unit cost data that were sourced from the literature were converted to 2016 US$ with purchasing power parity.^19^

### Model outputs and sensitivity analyses

The main outputs of the cost-effectiveness model are average per-patient costs, life-years, and QALYs for treatment of post-partum haemorrhage with and without tranexamic acid and calculation of the incremental cost-effectiveness ratio (ICER), which is the ratio of the difference in costs between treatment strategies to the difference in QALYs. The resulting ICER can be compared to a country-specific cost-effectiveness threshold value; an ICER that falls below the threshold value would generally be considered cost-effective. We adopted a range of cost-effectiveness threshold values in each country ($446–$2880 per QALY in Nigeria and $314–$2416 per QALY in Pakistan), which were obtained from an analysis by Woods and colleagues.^20^ Their calculation of cost-effectiveness thresholds is based on the premise that health-care budgets are constrained; if an intervention offers health gains but also incurs additional costs, then the decision to fund the intervention should be informed by the value of the other interventions that must be foregone. The cost-effectiveness threshold ranges estimated by Woods and colleagues are based on an understanding of the association between changes in health-care expenditure and health outcomes in the National Health Service (NHS) in England, which was then applied to other countries with different income levels by use of estimates of the income elasticity of the value of health.^20^

To address uncertainty in the data inputs for the cost-effectiveness model, we did a series of one-way sensitivity analyses varying one parameter at a time and observing the effect on the ICER. All parameters listed in Table 1, Table 2, Table 3 were varied across the ranges specified, with the exception of the administration cost of tranexamic acid. In the base case analysis, a discount rate of 3% per year was applied in the model but we explored the effect of varying this rate from 1·5% to 10% in one-way sensitivity analyses.^21^ The reason for discounting in a cost-effectiveness analysis is to reflect the fact that costs and health benefits in the present are valued more highly than costs and health benefits occurring in the future.^22^

We also explored the combined effect of parameter uncertainty on the ICER by simultaneously sampling input values across multiple parameters by use of probabilistic sensitivity analysis.^23^ We assigned distributions for parameter estimates derived from the WOMAN trial and health-state utility values from the literature (Table 1, Table 2) and used Monte Carlo simulation to draw 10 000 samples across all distributions. We present the results with a cost-effectiveness acceptability curve to show the probability that treatment of post-partum haemorrhage with tranexamic acid is cost-effective across a range of threshold values in each country.^20^

### Role of the funding source

The funders of this study had no role in the study design, data collection, data interpretation, data analysis, or writing of this report. The authors had full access to all the data and had final responsibility for the decision to submit for publication.

## Results

Average costs, life-years, and QALYs per patient for treatment of post-partum haemorrhage with and without tranexamic acid in Nigeria and Pakistan are summarised in table 4. Although the effect of tranexamic acid in reducing the risk of death due to bleeding in the model is the same in both countries, we observed differences in survival and QALYs because the baseline probabilities of death due to bleeding and post-discharge life expectancies differ between the two countries. Table 4 also summarises the base-case point estimates for the ICERs and the country-specific threshold ranges. The ICER estimates are below the lower bounds of the cost-effectiveness threshold ranges, which suggests that treatment of post-partum haemorrhage with tranexamic acid is cost-effective in both countries.

**Table 4:** Average costs, life-years, and QALYs per patient with and without tranexamic acid for the treatment of post-partum haemorrhage as well as base case ICERs in each country

	**Cost (US$)**	**Life-years**	**QALYs**	**ICER**	Cost-effectiveness threshold range*
**Nigeria**					
Tranexamic acid	127·18	22·13	20·58	··	··
No tranexamic acid	90·06	21·94	20·40	··	··
Difference	37·12	0·19	0·18	$208 per QALY	$446–$2880 per QALY
**Pakistan**					
Tranexamic acid	118·03	24·59	22·86	··	··
No tranexamic acid	111·48	24·50	22·78	··	··
Difference	6·55	0·09	0·08	$83 per QALY	$314–$2416 per QALY

QALY=quality-adjusted life-year. ICER=incremental cost-effectiveness ratio.

* Values were adjusted for purchasing power parity.^20^

**Figure 2:**
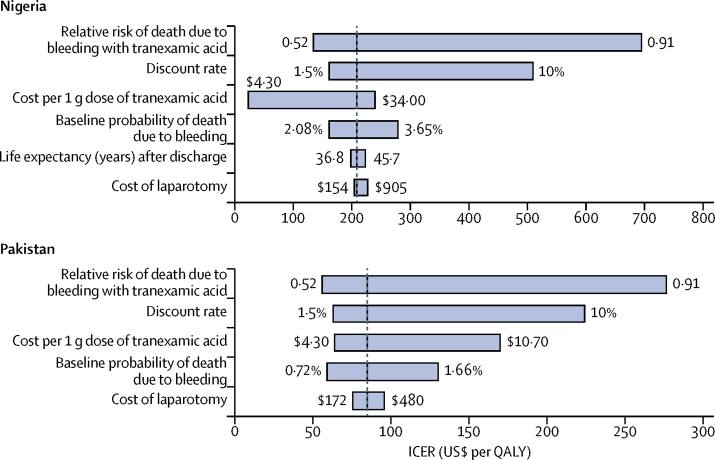
Tornado diagram showing the effect of varying each parameter on its own on the ICER in Nigeria and Pakistan Only parameters that resulted in a difference of more than $20 per quality-adjusted life-year (QALY) in the incremental cost-effectiveness ratio (ICER) when varied between the lower and upper bounds of the plausible ranges are shown. The vertical line indicates the base case estimate of the ICER. The cost-effectiveness threshold range is $446–$2880 per QALY in Nigeria and $314–$2416 per QALY in Pakistan.

We did a series of one-way sensitivity analyses to explore the effect of varying different parameters across a plausible range of values on the ICER. Figure 2 shows those parameters that led to a change in the ICER of greater than $20 per QALY when inputs were varied from the lowest to the highest values over the specified ranges. In both countries, the ICER was shown to be most sensitive to variations in the relative risk of death due to bleeding with tranexamic acid, the discount rate, the cost of tranexamic acid, and the baseline probability of death due to bleeding. In Pakistan, when the relative risk of death due to bleeding with tranexamic acid was increased to 0·91, the ICER remained below the lower bound of the cost-effectiveness threshold of $314 per QALY. In Nigeria, increasing the relative risk of death due to bleeding with tranexamic acid to the upper bound of 0·91 resulted in an ICER of $692 per QALY, while increasing the discount rate to 10% resulted in an ICER of $507 per QALY. These ICERs are still within the cost-effectiveness threshold range for Nigeria ($446–$2880 per QALY). This analysis suggests that the cost-effectiveness results are robust to the uncertainty surrounding the value of the inputs for the key parameters in the model.

**Figure 3:**
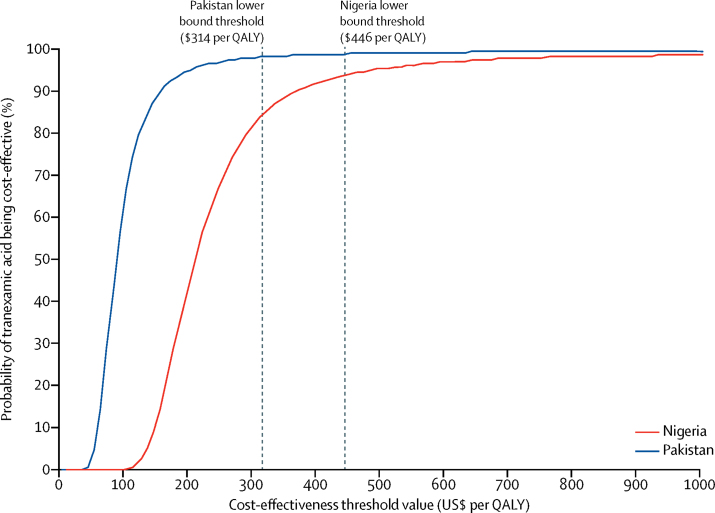
Cost-effectiveness acceptability curves showing the probability that tranexamic acid is cost-effective across a range of threshold values in Nigeria and Pakistan QALY=quality-adjusted life-year.

Figure 3 shows the effect of simultaneously varying the value of multiple parameters in the cost-effectiveness model on the ICER in each country. The vertical axis indicates the probability that tranexamic acid is cost-effective at different threshold values on the horizontal axis. At the lower end of the threshold range for Pakistan ($314 per QALY), the probability that tranexamic acid is cost-effective is 98%. At the lower end of the threshold range for Nigeria ($446 per QALY), the probability that tranexamic acid is cost-effective is 93%.

## Discussion

Administration of tranexamic acid to women with post-partum haemorrhage has been shown to save lives when the drug is given within 3 h of giving birth,^5^ but this intervention incurs additional costs. We sought to evaluate whether the additional costs associated with routine use of tranexamic acid in women with post-partum haemorrhage represent an efficient use of scarce resources. We did this analysis in two countries with a high burden of maternal mortality, Nigeria and Pakistan, and found that tranexamic acid is likely to be highly cost-effective in both countries. Although this is the first analysis to evaluate the cost-effectiveness of tranexamic acid for treatment of post-partum haemorrhage, our findings are broadly consistent with those of previous economic evaluations that have shown the cost-effectiveness of tranexamic acid for treatment of excessive blood loss in other patient groups, including treatment of patients with bleeding trauma and those who have had elective surgery.^24^, ^25^, ^26^

As an inexpensive and potentially life-saving intervention, it is perhaps not surprising that tranexamic acid has been shown to be cost-effective in different settings, although uncertainty surrounding the price of the drug has been shown to have a substantial effect on ICER estimates in sensitivity analyses in our study and in previous studies.^24^, ^25^ Another important source of uncertainty in our cost-effectiveness analysis was the baseline probability of death due to bleeding in each country. We chose to evaluate the cost-effectiveness of tranexamic acid in countries with high maternal mortality as this is the setting where we expect to see the largest benefit in terms of a reduction in the number of deaths due to bleeding. Caution should be exercised in generalisation of the cost-effectiveness results reported here to countries with much lower baseline rates of maternal mortality due to post-partum haemorrhage. It is also important to consider the generalisability of the results of this analysis across different settings within Nigeria and Pakistan, given the realities of health-care provision in both countries. We have evaluated the cost-effectiveness of tranexamic acid when given within 3 h of birth, which requires timely access to skilled health personnel. According to estimates from 2012, the proportion of births attended by skilled health personnel was 56% in Pakistan.^27^ In Nigeria, where there are substantial within-country socioeconomic inequalities in access to maternal health care, the proportion was 40%.^27^, ^28^

Our cost-effectiveness analysis has a number of potential limitations. The maximum period of follow-up in the WOMAN trial was 42 days and therefore in the cost-effectiveness model it was necessary to make a general assumption about long-term survival beyond the trial period. We based our assumption on country-specific average estimates of age-adjusted female life expectancy from the literature.^9^ This assumption meant that the time horizon of the model for patients who were alive at discharge was quite long, making the results sensitive to variations in the discount rate. For this reason, we tested the effect of increasing the discount rate up to 10% and found that tranexamic acid remained cost-effective in one-way sensitivity analysis. Another limitation of the short follow-up period is that we were unable to collect any data on wider health benefits beyond the immediate survival of the mother. For example, we did not attempt to measure or quantify the effects of maternal survival on the survival or quality of life of the newborn child or the family, thereby potentially underestimating the benefits of tranexamic acid.

To use the results of any cost-effectiveness analysis to inform decision making, it is necessary to compare the ICER generated by the model to a threshold value. Various different approaches have been used to estimate cost-effectiveness thresholds.^20^ Historically, the ICERs for health interventions in low-income and middle-income countries have been compared to a threshold value of one to three times the country's gross domestic product (GDP) per capita.^20^, ^29^ This approach for estimation of the threshold is conceptually linked to how individuals value health and their willingness to pay for additional health benefits, but it has been criticised because this measure is not directly linked to an assessment of the value of a new intervention in relation to what other health-generating interventions would need to be displaced.^20^, ^29^, ^30^ In this study, we chose to compare the ICER results to cost-effectiveness threshold ranges that were estimated on the basis of the opportunity costs of health-care spending that better reflect the constrained nature of health-care budgets. The thresholds we used are lower and therefore more stringent than thresholds based on GDP per capita. This approach gives us further confidence in our conclusion that early administration of tranexamic acid is cost-effective for the treatment of post-partum haemorrhage in Nigeria and Pakistan.
