# Supplementary material for: Tranexamic acid for treatment of women with post-partum haemorrhage in Nigeria and Pakistan: a cost-effectiveness analysis of data from the WOMAN trial
Source: Lancet Glob Health. 2018 Jan 19;6(2):e222–8. doi: 10.1016/S2214-109X(17)30467-9 (PMC5785366; doi:10.1016/S2214-109X(17)30467-9)
Supplement: Supplementary appendix [file mmc1.pdf]

THE LANCET
Global Health

Supplementary appendix

This appendix formed part of the original submission and has been peer reviewed. We post it as supplied by the authors.

Supplement to: Li B, Miners A, Shakur H, Roberts I, on behalf of the WOMAN Trial Collaborators. Tranexamic acid for treatment of women with post-partum haemorrhage in Nigeria and Pakistan: a cost-effectiveness analysis of data from the WOMAN trial. *Lancet Glob Health* 2018; **6:** e222–28.

Supplementary appendix

Number of ICU days and length of stay for patients who received treatment within 3 hours of giving birth in the WOMAN trial showing no difference between arms

	TXA	No TXA	p value
ICU days			
n	7517	7407	
Mean (SD)	0·18 (1·13)	0·17 (0·93)	0·772
Median (IQR)	0 (0-0)	0 (0-0)	
Length of stay (randomisation to discharge in days)*			
n	7381	7244	
Mean (SD)	3·40 (3·70)	3·42 (3·88)	0·726
Median (IQR)	2 (1-4)	2 (1-4)	

*Excludes patients who died
